# Multiregional assessment of surface guidance for patient positioning in radiotherapy

**DOI:** 10.1016/j.tipsro.2026.100394

**Published:** 2026-03-25

**Authors:** Dávid Kanalas, Anna Jurczak

**Affiliations:** a Hochschule Campus Wien/University of Applied Sciences, Favoritenstraße 226, 1100 Vienna, Austria; b Klinik Donaustadt, Department of Radiation Oncology, Langobardenstraße 122, 1220 Vienna, Austria

**Keywords:** Radiotherapy, Patient positioning, Surface-guided radiotherapy, Setup accuracy, Treatment margins

## Abstract

Accurate and reproducible patient positioning is critical in external beam radiotherapy to ensure optimal target coverage while sparing surrounding healthy tissue. This retrospective single-institution study compared surface-guided radiation therapy (SGRT) using the Catalyst™ HD system with conventional skin-marker (SKM) alignment in thoracic and pelvic treatments. Sixty patients (20 thoracic, 40 pelvic) and 1,105 treatment fractions were analyzed. Thoracic patients received both SGRT and SKM workflows sequentially, enabling paired analysis; pelvic patients were treated using either SGRT or SKM exclusively. Translational 3D offsets, verified by image guidance, were quantified from couch shift data, and setup precision was evaluated using descriptive statistics, hypothesis testing, and linear mixed-effects models. In thoracic treatments, SGRT reduced mean 3D offsets from 0.91 cm to 0.42 cm (52 % reduction) and variance from 0.54 cm^2^ to 0.06 cm^2^ (p < 0.001). Pelvic SGRT cases showed a 37 % reduction in mean 3D offsets (0.68 cm to 0.43 cm) and variance reduction from 0.18 cm^2^ to 0.04 cm^2^ (p < 0.001). The proportion of fractions within 5 mm increased from 25.0 % to 65.2 % (thorax) and from 37.0 % to 67.7 % (pelvis) with SGRT. Setup margins calculated using the van Herk formula decreased from 1.27 cm to 0.46 cm (thorax) and from 0.92 cm to 0.35 cm (pelvis). These findings demonstrate that SGRT improves setup accuracy and reproducibility across anatomical sites, enabling tighter setup margins and supporting workflow efficiency, without reducing verification imaging frequency in IGRT-based treatments. Routine integration of SGRT can therefore enhance setup accuracy, improve setup precision, and support efficient positioning workflows in modern radiotherapy.

## Highlights

• A multi-regional analysis demonstrates improved positioning consistency in thoracic and pelvic radiotherapy.
• Large setup deviations were substantially reduced by SGRT compared with skin-marker–based alignment.
• SGRT increased the proportion of fractions within clinically acceptable setup tolerances.
• Setup-related margins were lower for SGRT across both anatomical regions.

## Graphical abstract

**Figure:**
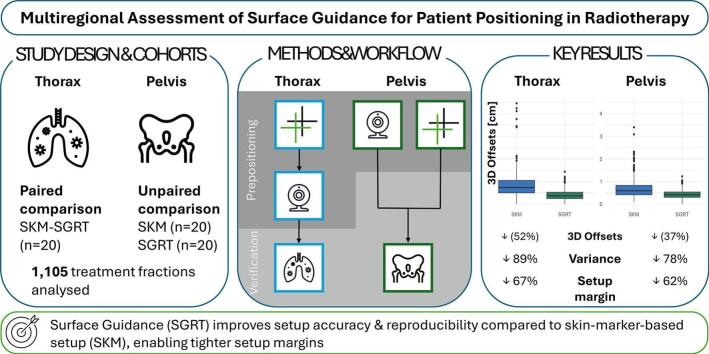

## Introduction

In external beam therapy accurate and reproducible patient positioning is essential. The goal is simple but critical: deliver the prescribed dose to the tumor while sparing surrounding healthy tissue. Small deviations from the planned position can lead to underdosage in the target or unnecessary radiation to organs at risk, thus impacting treatment efficacy and safety [1]. This is particularly challenging in regions where external geometry shows only weak correlation with internal anatomical position, such as the pelvis and thorax [2].

Historically, patient setup has relied on skin markers (SKM) in combination with in-room lasers, followed by imaging for verification [1]. While skin-marker-based positioning has been the clinical workhorse for decades, it is subject to limitations, including variability due to patient anatomy, fading of ink marks, inter-operator variability and dependence on subjective marking quality [3], [4]. These limitations extend beyond accuracy and affect clinical workflow. Surface-guided radiotherapy (SGRT) enables continuous, real-time monitoring of the patient’s surface before and during treatment. Using optical scanning for patient positioning, the system compares the live surface to a predefined reference, which may be derived either from the planning-CT (pCT) body contour or from a previously acquired surface scan, enabling early detection of deviations and reducing reliance on tattoos, skin markers or lasers. In contrast to conventional alignment based on three laser intersection points, SGRT employs thousands of surface points for registration, providing a substantially more robust geometric basis for patient setup [5].

Numerous studies have demonstrated the clinical benefits of SGRT across various anatomical sites. In addition to improving accuracy, surface guidance has been shown to support more reproducible patient positioning throughout the treatment course [1], [5], [6], [7], [8]. These advantages have also been demonstrated in thoracic, abdominal, and pelvic radiotherapy, where recent studies reported improved setup precision compared to conventional positioning [1], [7], [8]. Such benefits are particularly relevant in breast radiotherapy, where SGRT has been shown to reduce inter-fractional, intra-fractional, and inter-operator variability, thereby enhancing reproducibility [9]. SGRT detects surface changes arising from anatomical deformation or weight loss more readily than laser-based alignment, enabling early recognition of anatomical changes that may warrant further imaging assessment and consideration for treatment plan adaptation [10].

Despite the increasing implementation of SGRT in modern radiotherapy departments, evidence comparing its setup performance with conventional SKM-based patient positioning across multiple treatment regions and larger patient cohorts remains scarce. Most studies focus solely on breast cancer cases or are restricted to small sample sizes. Furthermore, few studies offer a direct comparison between SGRT-based and skin-marker-based positioning under routine clinical conditions.

Building on this, the present study compares the positioning accuracy and precision of surface guided and skin-marker-based setup in thoracic and pelvic cancer patients. The primary goal is to quantify differences in setup accuracy (systematic error, Σ) and precision (random error, reproducibility, σ), and to evaluate the potential of SGRT integration into routine workflows. It was hypothesized that SGRT would result in significantly reduced setup deviations and lower variability compared to conventional SKM-based alignment.

## Materials and methods

This retrospective observational study was conducted at the Department of Radiation Oncology, Klinik Donaustadt (Vienna, Austria) and approved by the Ethics Committee of University of Applied Sciences (Reference No. EK-273/2025, 30 June 2025). Between February and September 2023, sixty patients (40 pelvic, 20 thoracic) treated with external beam radiotherapy (volumetric modulated arc therapy or 3D conformal two-field techniques) were included. All eligible patients treated during the study period were included. No fractions were excluded due to missing verification imaging. In every thoracic patient, the first fraction was excluded because it followed a simulation workflow with SKM-based positioning and therefore did not provide a comparable SGRT reference. In total, 20 thoracic fractions (one per patient) were excluded.

Patient treatments were delivered on three linear accelerators. Two systems were equipped with Catalyst™ HD surface guidance, and one system used Catalyst+™ HD (C-RAD AB, Uppsala, Sweden). No system recalibration or hardware modifications occurred during the study period. While the Catalyst™ system does not allow defining a traditional region of interest, the system uses a predefined bounding box. The reference surface was restricted by adjusting the bounding box to exclude deformable anatomy. In thoracic patients, areas such as the chin, neck, and upper abdomen were excluded. For pelvic cases, lower abdomen and genital region were omitted, restricting the matching surface to external regions overlying relatively rigid anatomy. For thoracic patients, the SGRT reference surface was initially obtained using a Sentinel 4DCT (C-RAD AB, Uppsala, Sweden) scan acquired during CT simulation. The Sentinel 4DCT-derived surface provided a sufficient and anatomically accurate reference for simulation. However, the Sentinel system employs a single-camera configuration, which limits surface coverage compared to the three-camera configuration of the Catalyst™ HD system used during treatment. To take advantage of the improved surface coverage and registration provided by the three-camera system, a new SGRT reference surface was created directly on the treatment machine after the first fraction. In contrast, pelvic patients used a reference surface derived from the pCT body contour.

The thoracic subgroup comprised 164 paired fractions. The initial fraction, which relied on a single-camera reference surface, was excluded from analysis. The pelvic subgroup comprised 505 SKM fractions and 436 SGRT fractions from independent patient cohorts. For pelvic cases, assignment to SKM or SGRT reflected routine clinical workflows and was not randomized. Allocation followed clinical routine and system availability during SGRT implementation. All data were collected from routine clinical practice, without influencing treatment delivery or patient management, and were pseudonymized in compliance with local data protection laws.

Thoracic patients were positioned in a supine, head-first orientation using IT-V (Innovative Technologie Völp e.U., Innsbruck, Austria) BreastSTEP™ immobilization system. Pelvic patients were positioned using the indexed IT-V ProSTEP™ immobilization device.

**Fig. 1:**
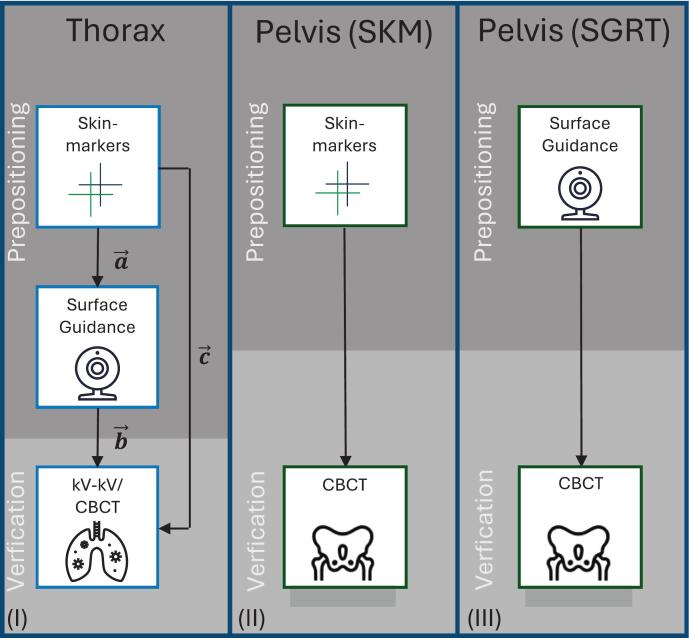
Patient setup workflows: All translational offsets refer to the PTV-centered isocenter defined in the planning CT. (I) Thoracic setup with SKM and SGRT. (II) Pelvic setup with SKM only. (III) Pelvic setup with SGRT only.

For thoracic treatments, both setup methods (SKM and SGRT) were applied sequentially within the same patients, as illustrated in Fig. 1(I). Initial patient alignment was performed using skin markers and room lasers, followed by surface-guided setup correction with the Catalyst™ system. Image-guided verification (CBCT or kV-kV) was performed only after both setup steps, ensuring that the positioning workflow remained non-ionizing until final verification. In contrast, pelvic patients underwent either SKM-based setup (Fig. 1(II)) or SGRT-based (Fig. 1(III)), depending on cohort assignment. To account for this, pelvic data were analyzed using unpaired statistical tests, and the interpretation of findings explicitly acknowledges that inter-individual anatomical differences may contribute to the observed effect sizes.

For the thorax subgroup initial alignment was performed using SKM and room lasers (Apollo, LAP, Germany), followed by a surface-guided correction using the Catalyst™ HD system. The Catalyst™ HD system was operated in its standard three-camera configuration. The PTV-centered isocenter from the planning CT served as a fixed reference for setup, surface registration, and verification. Translational setup corrections were recorded along the lateral (x), longitudinal (y), and vertical (z) axes, and total 3D offsets were derived using the Euclidean norm. For exploratory purposes, axis-specific descriptive statistics of translational components (x, y, z) were also computed; these data are provided in Supplementary Tables S 1, S 2 (details in Supplementary Material). Verification was performed using image registration with cone-beam CT (CBCT) or kV-kV imaging, conducted by radiation therapists (RTTs). Thoracic cases were predominantly verified using CBCT, with kV-kV imaging applied in two patients, and registrations were based on bony anatomy according to institutional protocol. Pelvic cases underwent CBCT verification exclusively, with image matching performed using soft-tissue alignment.

Geometric alignment between the SGRT systems and the imaging isocenters (kV-kV/CBCT) was verified through routine departmental quality assurance, including daily positional constancy checks and monthly end to end tests with tolerance limits of ≤ 1 mm. These checks served to confirm system stability; no systematic drift or recalibration-related changes were observed during the study period. For each treatment fraction all treatment couch shifts were automatically recorded within the record-and-verify system Mosaiq® (Elekta AB, Stockholm, Sweden). Setup accuracy was assessed in both thoracic and pelvic subgroups by comparing 3D offsets between surface-guided and skin-marker-based positioning. Descriptive statistics (mean, standard deviation, variance, 95th/99th percentiles, and proportion of offsets ≤ 5 mm) were calculated for each method and region. Setup accuracy was defined as the systematic deviation (Σ) of the pre-imaging patient setup relative to the imaging isocenter (kV-kV/CBCT), whereas precision was defined as the random variability (σ) of repeated daily setups around this mean position. Taken together, these two components describe the overall setup uncertainty. Hypothesis testing included paired t-tests for thoracic cases (where both methods were applied within the same patients) and Welch-corrected unpaired t-tests for pelvic cases (where independent patient groups were compared). Normality was verified using Shapiro-Wilk tests for thoracic paired differences and separately for each pelvic method, and F-tests were performed to compare variances. Linear mixed-effects models were fitted using the *lme4* package with *lmerTest* for p-values to evaluate the effects of setup method and treatment fraction. In the thoracic cohort, repeated measurements within patients were accounted for using random intercepts (3D_offset ∼ Method × Fraction + (1 | Patient)). In the pelvic cohort, SGRT and SKM represented independent patient groups. Therefore, fraction-related trends were analyzed using a fixed-effects model without random terms, including Method, Fraction, and their interaction (3D_offset ∼ Method × Fraction). Margins were calculated separately for each anatomical region and setup method from systematic and random errors using the van Herk formula [11] and are reported as isotropic setup margins. These margins quantify the setup-related uncertainty prior to image-guided correction and therefore represent a margin contribution additional to IGRT-based margins. In addition to the isotropic margins used for the analysis, axis-specific evaluations were performed, including descriptive per-axis offsets (mean and SD) as well as anisotropic setup margins calculated separately for the lateral (x), longitudinal (y), and vertical (z) directions; these results are reported in Supplementary Tables S 1-S 4 (details in Supplementary Material).

All calculations were performed using Microsoft Excel® (Microsoft Corporation, Redmond, Washington, USA) Version 2505 Build 16.0.18827.20102 and R (The R Foundation for Statistical Computing, Vienna, Austria), version 4.3.2, RStudio (Posit Software, PBC, Boston, MA, USA). A significance level of α = 0.05 was used throughout.

## Results

Results are reported separately for the thoracic and pelvic cohorts. The descriptive statistics for both positioning methods and anatomical regions are summarized in Table 1.

**Table 1:** Descriptive overview of setup accuracy by region, comparing surface-guided radiotherapy (SGRT) with conventional skin-marker (SKM) positioning. Metrics shown: mean 3D offset, variability, percentiles, proportion within 5 mm, and dominant deviation direction.

Metric	Thorax SKM	Thorax SGRT	*p*-val.	Pelvis SKM	Pelvis SGRT	*p*-val.
Mean 3D offset	0.89 cm	**0.42** cm	<0.001	0.68 cm	**0.43** cm	<0.001
Standard deviation	0.74 cm	**0.22** cm	<0.001	0.42 cm	**0.20** cm	<0.001
Variance	0.54 cm^2^	**0.06** cm^2^	<0.001	0.18 cm^2^	**0.04** cm^2^	<0.001
95th percentile	1.90 cm	**0.80** cm	<0.001	1.50 cm	**0.80** cm	<0.001
99th percentile	4.20 cm	**1.10** cm	<0.001	2.20 cm	**1.0** cm	<0.001
Proportion within 5 mm	25.0 %	**65.2** %	<0.001	36.6 %	**67.7** %	<0.001
Dominant deviation direction	z −0.28 cm	z −0.18 cm		x −0.35 cm	x −0.13 cm	

### Thoracic subgroup

In the thoracic cohort, SGRT significantly improved overall setup accuracy and precision compared to conventional skin-marker-based alignment. The mean 3D setup offset was 0.42 cm (95 % CI: 0.40–0.47) under SGRT, compared to 0.91 cm (95 % CI: 0.79–1.02) for skin markers. This corresponds to a 52 % reduction in mean offset. The linear mixed-effects model confirmed a method effect estimate of 0.47 cm (95 % CI: 0.150–0.301, *p* < 0.001), supporting a significant improvement with SGRT after controlling for repeated measures. The model-based estimate reflects the adjusted method effect after accounting for repeated measures and fraction trends and is therefore smaller than the raw mean difference of 0.47 cm derived from unadjusted group averages.

**Fig. 2:**
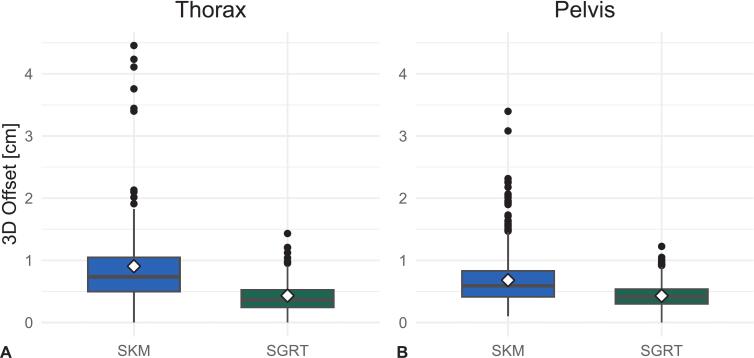
3D setup offsets for SGRT and SKM in thoracic (A) and pelvic (B) treatments; identical y-axis scaling for comparison. Boxes represent the interquartile range with median lines; whiskers extend to 1.5 × IQR. Black dots indicate outliers, and white diamonds mark mean values.

The distribution of 3D offsets (Fig. 2(A)) shows consistently lower medians and a narrower interquartile range for SGRT compared to SKM, reflecting improved precision. A significant reduction in variability was observed. The variance dropped from 0.54 cm^2^ (SKM) to 0.06 cm^2^ (SGRT), as confirmed by an F-test (F = 9.09, df = 163, *p* < 0.001). Percentile values and outlier occurrence followed the same pattern: the 95th and 99th percentiles were substantially lower under SGRT (0.90 cm and 1.20 cm, respectively) than with SKM (1.90 cm and 4.20 cm, respectively). Furthermore, 65.6 % of thoracic fractions under SGRT exhibited 3D offsets ≤ 5 mm, whereas only 26.0 % of skin-marker-based setups remained within this tolerance (Table 1).

**Fig. 3:**
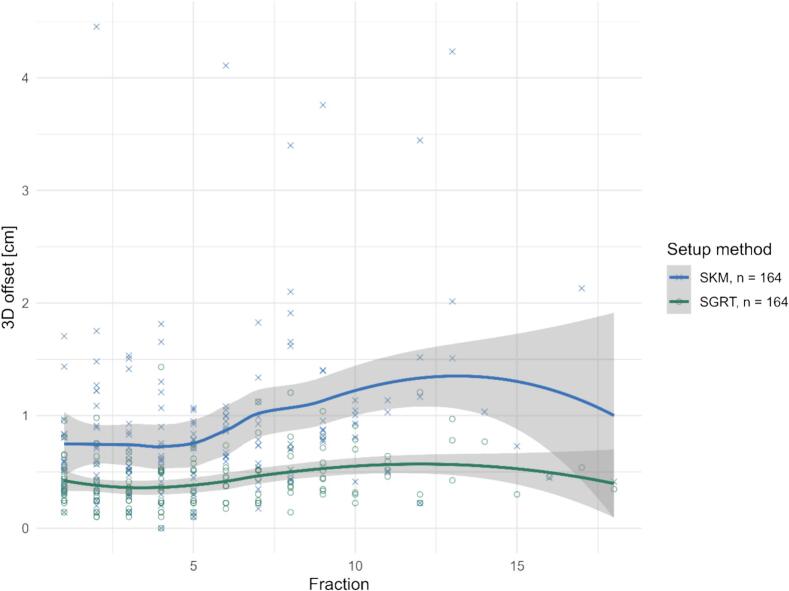
Fraction-wise 3D setup offsets in the thoracic cohort for SKM (× symbols) and SGRT (○ symbols). Each marker represents one treatment fraction. SGRT shows consistently lower median offsets and a narrower distribution across fractions compared with SKM. LOESS curves with 95 % confidence intervals illustrate the fraction-dependent trend for both positioning methods.

A trend analysis using the linear mixed-effects model showed a significant Fraction × Method interaction (*p* = 0.015). Offsets increased with fraction under SKM (β ≈ +0.051 cm/fraction, *p* < 0.001) but much less under SGRT (β ≈ +0.014 cm/fraction), i.e., SGRT’s advantage persisted and did not erode over the course (Fig. 3). The setup margin was substantially reduced with SGRT. For the thoracic subgroup, SGRT yielded a margin of 0.46 cm, compared to 1.27 cm with skin markers (Table 2).

**Table 2:** Mpop = mean population absolute 3D setup error; **Σ** = population systematic error; σ = population random error. All values correspond to the thoracic cohort and were calculated separately for SKM and SGRT. Isotropic setup margins for the thoracic patients were calculated separately for SKM and SGRT using pre-IGRT couch shifts and therefore represent setup margins only.

Method	Mpop [cm]	Σ [cm]	σ [cm]	Margin [cm]
SKM	0.89	0.37	0.50	1.28
SGRT	**0.42**	**0.14**	**0.17**	**0.46**

### Pelvic subgroup

In the pelvic subgroup, a comparable pattern was observed. SGRT reduced mean 3D setup offset to 0.43 cm (95 % CI: 0.41–0.45), compared to 0.68 cm (95 % CI: 0.64–0.72) with skin markers. A Welch-corrected *t*-test confirmed the statistical significance of this difference (*t* = 11.996, df = 731, *p* < 0.001). The linear mixed model estimated a method effect of 0.25 cm EMM difference (SKM-SGRT); model coefficient −0.230 cm (95 % CI: −0.379 to **−**0.081, p < 0.001), which was consistent with the descriptive results. Variability was significantly lower with SGRT: variance dropped from 0.18 cm^2^ (SKM) to 0.04 cm^2^ (SGRT), as confirmed by an F-test (F = 4.60, *p* < 0.001). The distribution of 3D offsets (Fig. 2(B)) confirms lower medians and reduced variability for SGRT compared to SKM, indicating improved setup consistency.

**Fig. 4:**
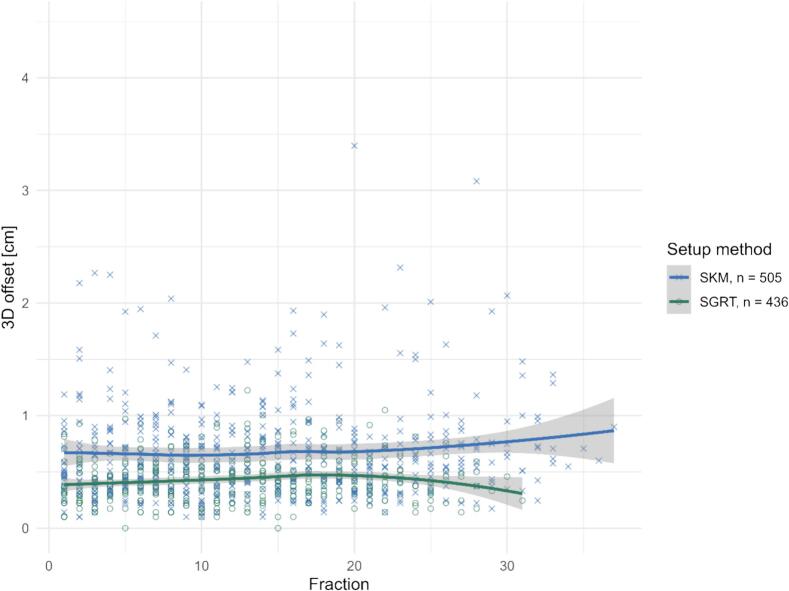
Fraction-wise 3D setup deviations in the pelvic subgroup for SKM (×) and SGRT (○). Each marker represents one treatment fraction. SGRT shows consistently lower median offsets and a narrower distribution across fractions compared with SKM. LOESS curves with 95 % confidence intervals illustrate the fraction-dependent trend for both methods.

The linear mixed model showed no significant Fraction × Method interaction (*p* = 0.432) but a small positive fraction effect across methods (β ≈ +0.004 cm/fraction, *p* = 0.004), consistent with a stable SGRT benefit over time (Fig. 4).

Setup margin analysis confirmed a reduction from 0.92 cm (SKM) to 0.35 cm (SGRT) in the pelvic group, supporting the feasibility of tighter setup margins for pelvic radiotherapy (Table 3).

**Table 3:** Mpop = mean population absolute 3D setup error; **Σ** = population systematic error; σ = population random error. All values correspond to the thoracic cohort and were calculated separately for SKM and SGRT. Isotropic setup margins for the pelvic patients were calculated separately for SKM and SGRT using pre-IGRT couch shifts and therefore represent setup margins only.

Method	Mpop [cm]	Σ [cm]	σ [cm]	Margin [cm]
SKM	0.68	0.29	0.27	0.92
SGRT	**0.43**	**0.09**	**0.18**	**0.35**

## Discussion

The results of this study demonstrate that SGRT using the Catalyst™ HD system provides significantly provides significantly improved setup performance (lower systematic and random error) compared to conventional skin-marker-based alignment in both thoracic and pelvic radiotherapy, supporting our initial hypothesis of improved accuracy and reduced variability. These findings are consistent with prior reports and reinforce the feasibility of integrating SGRT into routine clinical workflows across multiple anatomical regions [2], [9], [11].

Across the cohort, SGRT reduced mean 3D offsets by 52 % in thoracic cases (0.91 cm to 0.43 cm) and by 37 % in pelvic cases (0.68 cm to 0.43 cm). Standard deviation and variance were also significantly lower, indicating enhanced reproducibility and setup consistency. These improvements were both statistically significant and clinically relevant, particularly in the context of steep dose gradients used in modern conformal and modulated techniques.

Focusing on the thoracic subgroup, the most pronounced improvement occurred along the vertical (z) axis − a direction particularly sensitive to respiratory motion [12]. Axis-specific translational statistics for the thoracic subgroup are provided in Supplementary Table S 1 and reflect the vertical-axis predominance observed in the main analysis. Our findings are consistent with those of González-Sanchis et al., who reported higher surface matching scores with SGRT (98.0 % vs. 92.7 %) in 252 breast cancer patients across 1,170 fractions [9]. In our cohort, the proportion of fractions within 5 mm increased from 26.0 % (SKM) to 65.6 % (SGRT), a significant improvement despite different evaluation metrics. These results are also consistent with large-scale data from Haraldsson et al., who found SGRT reduced the proportion of fractions with setup errors > 5 mm to 1.7 %, compared with 27.5 % for conventional laser alignment, a pattern closely reflected in our thoracic results [13].

In the pelvic subgroup, the dominant deviation occurred laterally (x-axis), a direction often affected by pelvic soft-tissue variability and deformation [5], [11]. Corresponding axis-specific translational statistics for the pelvic subgroup are reported in Supplementary Table S 2 and confirm the lateral/vertical directional patterns identified in the primary analysis. SGRT reduced this deviation from −0.35 cm to −0.13 cm and improved overall setup consistency, lowering the mean 3D offset by 37 % and increasing the proportion of fractions within 5 mm from 36.6 % (SKM) to 67.7 % (SGRT). These results compare favorably with those of Mannerberg et al., who observed smaller but significant gains in ultra-hypofractionated prostate cancer patients, including reductions in lateral (0.19 cm to 0.11 cm) and vertical (0.26 cm to 0.22 cm) deviations [7]. In our dataset, the lateral axis improvement was more pronounced at 0.22 cm (−0.35 cm to −0.13 cm), underscoring that SGRT’s benefits extend beyond breast and thoracic radiotherapy to pelvic treatments, particularly when immobilization challenges and soft-tissue variability are effectively managed.

Beyond positional accuracy, SGRT enabled the early detection of misalignments caused by incorrect isocenter placement or misapplied immobilization devices, allowing corrective action before beam delivery. This observation was made by the RTTs during routine setup and was not quantitatively recorded in our dataset. Such errors might have remained undetected in an SKM-based workflow until verification imaging. This aligns with findings by Al-Hallaq et al., who highlighted SGRT’s role in improving patient safety through early detection of incorrect isocenter or immobilization [1].

In contrast, several studies have reported more modest improvements with SGRT. Naidoo and Leech described only limited advantages of SGRT over tattoo-based workflows in breast radiotherapy, attributing this to respiratory motion, soft-tissue deformation, smaller ROIs and camera geometry constraints [14]. Similarly, Malhotra et al. observed that tattooless breast radiotherapy using AlignRT® achieved setup times that were only about 10–20 s faster than tattoo-based workflows and did not significantly reduce pre-treatment imaging shifts, indicating non–inferior but only marginally superior positional accuracy [15]. Such findings indicate that the magnitude of SGRT improvements depends strongly on ROI stability, scan range, immobilization and system configuration.

Taken together, these findings confirm and extend the results of multiple independent studies to a multi-regional clinical cohort. In this analysis, setup margins were calculated as isotropic margins, derived from the isotropic systematic (Σ) and random (σ) components of the 3D translational vector using the van Herk formula [16]. SGRT reduced isotropic margins from 1.28 cm to 0.46 cm in the thoracic group and from 0.92 cm to 0.35 cm in the pelvic group, demonstrating its potential to support tighter setup margins. At the same time, SGRT primarily improves pre-imaging setup consistency and therefore complements IGRT-based workflows. Because daily IGRT substantially reduces residual positioning error, the margins reported here describe the setup uncertainty and should be interpreted as an additional contribution to, rather than a replacement for, IGRT-based margins. Typical IGRT-based margins reported in the literature range from 3 to 5 mm, depending on anatomical site and protocol [11]. IGRT-specific margins were not calculated in this study because residual post-correction errors were not systematically recorded.

While the results are compelling, the study’s hybrid design warrants consideration. For thoracic patients, SGRT and SKM setups were applied sequentially within the same individuals, enabling a paired comparison and controlling for confounders such as BMI, breast size, and immobilization technique. In the pelvic subgroup, however, SGRT and SKM were evaluated in separate patient groups, representing an unpaired analysis that may be influenced by inter-individual anatomical variability.

Limitations include retrospective study design, single institution data and no analysis of rotational errors, which represents a methodological limitation − particularly in pelvic treatments, where rotations may contribute to clinically relevant misalignments [13]. The non-randomized workflow-based allocation in the pelvic cohort may introduce allocation bias, which was considered when interpreting between-group differences. All eligible patients treated within the study period were included to avoid sampling bias, although allocation to SKM or SGRT was determined by clinical workflow rather than randomization. As SGRT relies exclusively on the external surface, concordance between surface alignment and internal anatomy cannot always be assumed, particularly in regions with substantial soft-tissue variability [16]. SGRT also has the potential to improve inter- and intra-fractional control and to support six-degree-of-freedom (6DoF) corrections when used with suitable treatment couches, although these aspects were not evaluated in this study. Operational limitations should also be considered, including occasional camera occlusion and the constraints of the Catalyst™ bounding-box–based surface selection. Intra-fraction variability, influenced by patient movement and operator-dependent imaging and matching, represents an additional limitation. All verification procedures were performed by RTTs under routine clinical conditions, ensuring standardized practice but not eliminating residual variability. Despite these constraints, the study offers practical insights into integration of SGRT into routine workflows for thoracic and pelvis radiotherapy.

## Conclusion

SGRT significantly improved setup accuracy and precision compared with skin-marker-based positioning in both thoracic and pelvic radiotherapy. Although this study focused exclusively on inter-fraction translational setup variation, SGRT is also known to enhance intra-fractional stability and supports 6DoF corrections when used in combination with appropriate treatment couches. At the same time, important operational limitations must be considered, including camera occlusion, dependence on the selected surface, and the fact that external surface alignment does not fully correspond to internal anatomy. Overall, SGRT enhances pre-imaging setup consistency and complements IGRT-based workflows, without reducing the requirement for verification imaging.

## Declaration of generative AI in scientific writing

During the preparation of this work the authors used ChatGPT (OpenAI, San Francisco, CA, USA) in order to refine and optimize R code for statistical analysis and data visualization, as well as to check grammar and improve the readability and language of the manuscript. After using this tool/service, the authors reviewed and edited the content as needed and take full responsibility for the content of the published article.

## Declaration of competing interest

The authors declare that they have no known competing financial interests or personal relationships that could have appeared to influence the work reported in this paper.
