# Supplementary material for: Multiregional assessment of surface guidance for patient positioning in radiotherapy
Source: Tech Innov Patient Support Radiat Oncol. 2026 Mar 25;38:100394. doi: 10.1016/j.tipsro.2026.100394 (PMC13054430; doi:10.1016/j.tipsro.2026.100394)
Supplement: Supplementary Data 1 [file mmc1.docx]

# Supplementary Materials

Translational setup corrections were recorded in three spatial axes: longitudinal (y: superior–inferior), lateral (x: right–left), and vertical (z: anterior–posterior). For each fraction, the following vectors were defined:

The total deviation vector was defined as c⃗=a⃗+b⃗, where a⃗ represents the initial shift from skin-marker-based setup to surface guided correction, and b⃗ the residual deviation from SG position to kV-kV/CBCT-verified alignment.

For each fraction, the magnitude of total offset $\left| c \right|$ was calculated using the Euclidean Norm:

$${Offset}_{3D}=\sqrt{x^{2}+y^{2}+z^{2}}$$

Where x, y, z represent translational components along the lateral, longitudinal, and vertical axes, respectively. This approach provides a scalar measure of the combined displacement in all three axes for each fraction.

To estimate safety margins, we used the van Herk formula [11]:

$$Margin=2.5\times\Sigma+0.7\times\sigma$$

Where, $\Sigma$ represents the standard deviation of patient-specific means (systematic error), and $\sigma$ the average within-patient standard deviation (random error) [13]. Setup margins were derived isotropically from the 3D vector magnitude of the setup error. Margins were calculated separately for each anatomical region and setup method.

This section provides axis-specific descriptive offsets (Supplementary Tables S1-S2) and anisotropic setup margins calculated separately for the lateral, longitudinal, and vertical axes (Supplementary Tables S3-S4).

| **Setup method** | **Mean x [cm]** | **SD x [cm]** | **Mean y [cm]** | **SD y [cm]** | **Mean z [cm]** | **SD z [cm]** |
| --- | --- | --- | --- | --- | --- | --- |
| SKM | −0.05 | 0.82 | −0.09 | 0.51 | −0.28 | 0.59 |
| SGRT | −0.02 | 0.25 | −0.002 | 0.26 | −0.13 | 0.27 |

**Supplementary Table S 1: Thorax: Axis-specific offsets (mean and SD per method).**

| **Setup method** | **Mean x [cm]** | **SD x [cm]** | **Mean y [cm]** | **SD y [cm]** | **Mean z [cm]** | **SD z [cm]** |
| --- | --- | --- | --- | --- | --- | --- |
| SKM | −0.04 | 0.55 | 0.07 | 0.36 | 0.10 | 0.44 |
| SGRT | −0.08 | 1.14 | 0.13 | 1.89 | −0.28 | 1.01 |

**Supplementary Table S 2: Pelvis: Axis-specific offsets (mean and SD per method).**

| **Setup method** | **Axis** | **Mpop [cm]** | **Σ [cm]** | **σ [cm]** | **Margin [cm]** |
| --- | --- | --- | --- | --- | --- |
| SKM | Lateral (x) | −0.04 | 0.55 | 0.51 | 1.72 |
| SKM | Longitudinal (y) | −0.05 | 0.30 | 0.33 | 0.99 |
| SKM | Vertical (z) | −0.22 | 0.38 | 0.43 | 1.25 |
| SGRT | Lateral (x) | −0.005 | 0.18 | 0.17 | 0.57 |
| SGRT | Longitudinal (y) | 0.01 | 0.14 | 0.20 | 0.49 |
| SGRT | Vertical (z) | −0.09 | 0.19 | 0.19 | 0.62 |

**Supplementary Table S 3: Anisotropic setup margins per axis and setup method for thoracic cohort.**

| **Setup method** | **Axis** | **Mpop [cm]** | **Σ [cm]** | **σ [cm]** | **Margin [cm]** |
| --- | --- | --- | --- | --- | --- |
| SKM | Lateral (x) | −0.07 | 0.35 | 0.38 | 1.13 |
| SKM | Longitudinal (y) | 0.06 | 0.27 | 0.23 | 0.82 |
| SKM | Vertical (z) | 0.11 | 0.29 | 0.33 | 0.95 |
| SGRT | Lateral (x) | −0.07 | 1.06 | 1.06 | 1.79 |
| SGRT | Longitudinal (y) | 0.14 | 0.90 | 1.64 | 3.40 |
| SGRT | Vertical (z) | −0.28 | 0.41 | 0.92 | 1.66 |

**Supplementary Table S 4:** **Anisotropic setup margins per axis and setup method for pelvic cohort.**
